# Palatability Assessment of Carbocysteine Oral Solution Strawberry Taste Versus Carbocysteine Oral Solution Mint Taste: A Blinded Randomized Study

**DOI:** 10.3389/fphar.2022.822086

**Published:** 2022-02-28

**Authors:** Yaguang Peng, Huan Zhang, Liucun Gao, Xiaoling Wang, Xiaoxia Peng

**Affiliations:** ^1^ Center for Clinical Epidemiology and Evidence-based Medicine, National Center for Children’s Health, Beijing Children’s Hospital, Capital Medical University, Beijing, China; ^2^ Department of Pharmacy, National Center for Children’s Health, Beijing Children’s Hospital, Capital Medical University, Beijing, China

**Keywords:** palatability, pediatric pharmaceutical products, taste assessment, carbocysteine, crossover

## Abstract

**Objective:** To compare and evaluate the palatability of two carbocysteine oral solutions (strawberry vs. mint taste) among healthy children aged 2–12 years.

**Methods:** A randomized, triple-blind, crossover, palatability trial in 42 children aged 2–12 years. All subjects received two preparations of carbocysteine oral solutions (strawberry vs. mint) according to randomized administration sequences, and the administration process was recorded by video. The palatability assessed by emotional valences was performed using a facial action coding system by FaceReader™, which reflected the quantification degree of emotion; a positive value represents positive emotion, and a negative value represents negative emotion. At the same time, a face-to-face interview was conducted for 5- to 12-year-old participants. Then, the taste preferential rates were compared to assess the palatability of two carbocysteine oral solutions.

**Results:** Forty-two children were enrolled in this study. Twenty children first tasted the carbocysteine oral solution mint taste and then the strawberry taste preparation (M-S sequence), while 22 children tasted the strawberry preparation first and then the mint one (S-M sequence). The emotional valence of mint preparation (−0.9 in M-S and −1.2 in S-M) was both relatively lower than that of strawberry taste (both −0.7 in M-S and S-M) in two sequences; 69.0% (29/42) of participants’ emotional valences for strawberry preparation were higher than those for mint preparation. Among 27 participants aged ≥5 years, the taste preference rate was 88.5% (23/26) for the strawberry preparation (one missing value for the taste preference), and 77.8% of participants (21/27) chose the strawberry preparation if they had to take the medicine one more time.

**Conclusion:** The carbocysteine oral solution with strawberry taste is an appealing preparation since it was better received by children. The facial action coding system could be an effective alternative for palatability assessment of pediatric pharmaceutical products.

## Introduction

The palatability of pediatric pharmaceutical products to children can influence medication compliance and also profoundly impacts the resulting therapeutic outcome (Thompson et al., 2015; Venables et al., 2015; Ternik et al., 2018). Children have a highly developed perception system for taste, smell, or chemical stimuli. The rejection of unpalatable pharmaceutical products is a basic biological reflex, which may lead to decreased medication compliance and inaccurate or even harmful dosage, thus hampering the ideal treatment efficacy. Studies have found that more than 50% of children aged less than 6 years had difficulty using pharmaceutical products (Shimokawa et al., 2009); the potential factors resulting in this include how easy it is to swallow, formulation, size and shape, and factors related to palatability such as taste, flavor, and smell (Forough et al., 2018; Min et al., 2019; Gleeson et al., 2021). Therefore, better palatability of pharmaceutical preparations is pivotal in improving medication compliance in pediatric patients (Kim et al., 2020; Lopalco and Denora, 2020; Moreira and Sarraguça, 2020). With the advancement in taste-modifying techniques in food and pharmaceutical industries, the assessment of palatability, typically done with *in vivo* and *in vitro* evaluations, is becoming increasingly important (Forough et al., 2018). Several instruments and tools have been developed and applied for palatability assessment, such as a taste panel (Truong et al., 2021), visual analog scale (Tanaka et al., 2020a), facial hedonic scale (Salman et al., 2018a), and an electronic tongue (Wang et al., 2021). However, existing methodology and standards used for pediatric product palatability assessment are still scarce, making robust and consistent product evaluations difficult. Carbocysteine (S-carboxymethylcysteine or SCMC) is a mucoactive drug widely used in Europe, America, and Asia (Zeng et al., 2017). The anti-oxidative and anti-inflammatory action of carbocysteine plays an important role in chronic bronchitis and bronchial asthma for children. Carbocysteine is also one of the most commonly used medicines in pediatrics in China (Min et al., 2019), and children’s compliance is closely related to taste (Moreira and Sarraguça, 2020). Before 2019, the carbocysteine oral solution for pediatric use in China was only with mint taste (10 ml: 0.5 g), and a specific pharmaceutical product or dosage for children was not available. In clinical practice, the carbocysteine oral solution, which is known to have poor palatability, especially for young children, was administered in reduced doses according to age and weight. In 2019, a carbocysteine oral solution strawberry taste (100 ml: 2 g) that incorporated a flavor-masking technique was marketed in China. However, there is still a lack of evidence for the palatability of this oral solution. This study aimed to assess the palatability of two carbocysteine oral solutions, strawberry and mint tastes, for healthy children aged 2–12 years and explore the applicability of the potential tool, a facial action coding system, FaceReader™, for the palatability of pediatric pharmaceutical products to children.

## Methods

### Study Design and Participants

This randomized, triple-blind, crossover, palatability trial was conducted at a single center (Beijing Children’s Hospital, Beijing, China). Healthy children volunteers aged 2–12 years, who complied with the study instructions, were enrolled in the study. The inclusion criteria were as follows: (1) normal temperature, and (2) with no evident health issue. Exclusion criteria included the following: children who were unable to eat normally; those who suffer from dysphagia, gustatory dysfunction, or mental health disorders (which prevent them from expressing their taste); subjects who were allergic to carbocysteine or had an allergic predisposition; or subjects who were being treated with medications that might be incompatible with carbocysteine.

The difference of preference rate between the two carbocysteine preparations was applied to estimate the study’s sample size. The estimated rate for carbocysteine strawberry and mint tastes were 80% and 50%, respectively. According to PASS 16.0 software, the study needed 42 participants through the self-matching crossover design. With 42 participants, the power could reach 80% at the significance level of 0.05 without considering the subjects lost to follow-up. The study was registered online (Chinese Clinical Trial Registry, ChiCTR2100041778) and conformed to the ethical guidelines of the 1975 Declaration of Helsinki and was approved by the Institutional Review Board of Beijing Children’s Hospital (No. 2020-Z-170). The children and their legal representatives (parent or guardian) were fully informed of the study process *via* a short video of about 10 min and a detailed instruction by a researcher in order to fully understand the purpose, significance, and specific process of the study. Moreover, the whole process was completed with the assistance of two research assistants. Informed consent was obtained from each participant’s legal representative (parent or guardian) in the case of children aged 8 years or less. Additionally, it was asserted by the children themselves and consented by the legal guardian for children older than 8 years. The children were required to be awake before participating in the study. All the procedures were performed in the environment-friendly meeting room. The children were not given carbonated drinks, caffeinated drinks, grapefruit, lime, chocolate, and other fruits and food with stimulating taste 1 h before the trial. All the children avoided strenuous exercise and tried to keep their mood stable before taking the medicine.

### Randomization and Blinded Process

Participants were randomized to receive both the carbocysteine oral solutions strawberry and mint tastes using a triple-blinded crossover design. The administration sequence of two preparations was randomly blinded using SAS 9.4 and sealed in the envelopes for emergency unblinding. An independent clinical pharmacist left prescriptions in a dispensing room according to the blind code. Two milliliters of the two carbocysteine preparations were extracted from the original packaging using two 5-ml syringes with the needle removed and placed on the shelf according to the sequence number. The outer surfaces of the syringes were wrapped with opaque paper, which made it impossible to see the color and state of the carbocysteine in the syringes. Except for the clinical pharmacist who dispensed the carbocysteine preparations, the administration sequence was unknown to the investigator, the participants, and others involved in the data acquisition, including the interviewer, the data manager, and the statisticians.

### Administration Process

After eligibility for the study was confirmed, the enrolled children and their parents were taken to another private room one by one, asked to avoid communicating the taste of the preparations with each other. Firstly, the children were given three mouthfuls of room temperature pure water. Then, subjects received two single 2-ml doses of each carbocysteine formulation according to the randomized administration sequence. After tasting the first preparation, subjects cleansed their mouth again with several mouthfuls of water, waited for 10 min, and tasted the second preparation. The two preparations could be swallowed or vomited by participants autonomically. The carbocysteine oral solution strawberry taste was available in 100 ml: 2 g sugar-free solution by Beijing Chengji Pharmaceutical Co., Ltd., and the oral solution mint taste was available in 10 ml: 0.5 g sugar-free solution from Guangzhou Baiyunshan Pharmaceutical Holdings Co., Ltd. Guangzhou Baiyunshan Pharmaceutical General Factory. The entire process of medication administration was recorded, and the facial expressions during the two administration processes were collected by two cameras, placed in front of the participant, simultaneously for each child. Children under 2 years were assisted by a guardian during the administration process. All participants were asked to face the camera as much as possible while taking the medication.

### Palatability Assessment

A series of tools were applied to obtain a general evaluation of the palatability of two carbocysteine preparations, including facial action coding system, preferential and try-again method, and verbal response by interview.

**FIGURE 1:**
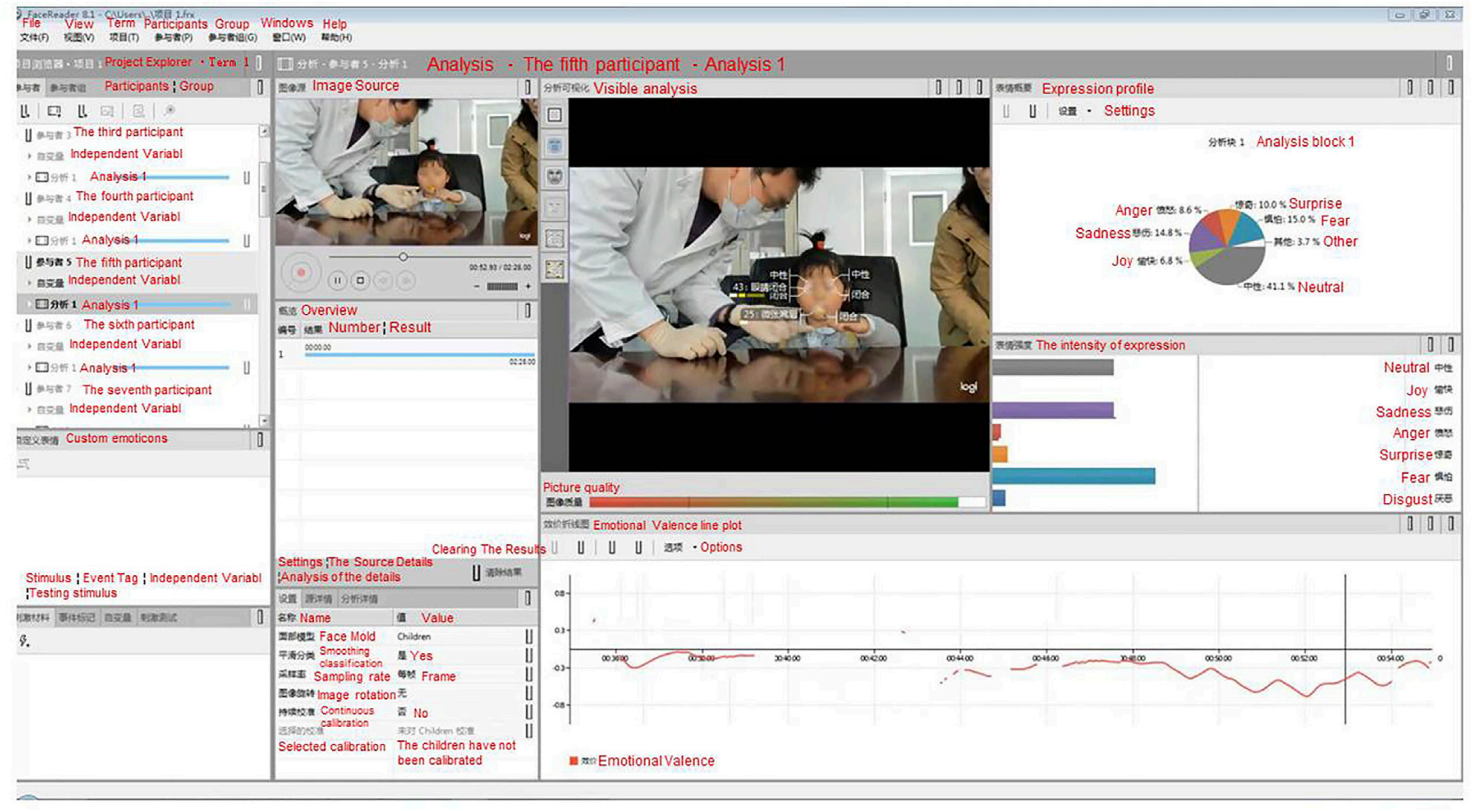
Example of the facial action coding system FaceReader™ (English translated from Chinese version) in this study that uses facial expression collection.

All the participant administration process videos were analyzed using a facial action coding system and professional analysis software, FaceReader™ (Noldus, Wageningen, Netherlands). Generally, human facial expressions can represent different moods or feelings, such as happiness, sadness, fear, disgust, surprise, anger, naturalness, and contempt. The software system analyzed the eight facial expressions above, together with emotional valence, arousal, and head orientation in complex environments based on deep neural network algorithms (shown in Figure 1). According to a recent validation study, FaceReader™ had the best performance among the major software tools for emotion classification currently available, with an average of 88% (Abdel-Rahman et al., 2021). As a “gold standard”, the consistency between the hedonic scales (5-point scales) and FaceReader™ was satisfied (*r* = 0.899) (Gadea et al., 2015). The tools had applied to psychological measurement for children and emotion recognition for adults in recent studies (Stöckli et al., 2018; Skiendziel et al., 2019).

After tasting both preparations, children, aged 5–12 years, were asked two questions: (1) “Now that you have tasted both medicines, which one of two medicines do you like?” (2) “If you had to take the medicine one more time, which one would you choose? The first one or the second one?” Participants responded by stating which of the preparations they preferred, and the interviewer recorded the child’s response. An interview was conducted to ask the children participants, aged 5–12 years, to describe the taste of the two preparations respectively after the administration process by an experienced interviewer. The interview was only performed for children over 5 years, mainly considering that children under 5 years old might not understand the interview questions clearly.

### Outcomes

The primary outcome was the mean emotional valence assessed by the facial action coding system for carbocysteine preparations in child participants. The emotional valence represents the degree of pleasure of the expression after taking the medicine, and a higher value corresponds to a higher degree of pleasure. The positive emotional valence indicated pleasure, while the negative suggested unpleasant emotions. The secondary outcomes were spitting out or non-ingestion of the two preparations during the administration process and the preference rates of taste and try-again choice by interview for children aged over 5 years.

### Safety and Tolerability

All adverse events, whether it was related to medication use or not, were recorded during the palatability assessment period and 24 h post-trial. The adverse events involved in this study mainly included hiccup and bucking due to swallowing of carbocysteine.

### Statistics

Data were depicted as a mean and standard deviation (SD) for continuous variables and as frequency (percentages) for categorical variables. The mean emotional valence of two preparations was described by median (percentile 25th and 75th) and compared by paired Wilcoxon signed rank test. The preferential analysis was analyzed by the Mainland-Gart test, an extension of the McNemar test that allows accounting for administration sequence effects (Becker and Balagtas, 1993). The exact probability method was used to calculate statistics for the comparison of rates differences between administration sequence groups in the crossover design. For the emotional valence in this crossover study, the sequence effect confounds with the carry-over effect. Hence, a hypothesis testing of no sequence effect is equivalent to a statistic test on the sum of the response variable (the test of carry-over effect) and to a statistic test on the difference of response variable (the test of treatment effect). The sum and difference of the emotional valence were computed and tested using the mixed effect model, which could analyze the interaction between the sequence of administration and the preference of the two preparations. A two-tailed *p* < 0.05 was considered significant. All statistics were conducted using SAS version 9.4 (SAS Institute, Cary, NC, United States) and R version 3.6.3 (R Foundation for Statistical Computing, Vienna, Austria).

## Results

**FIGURE 2:**
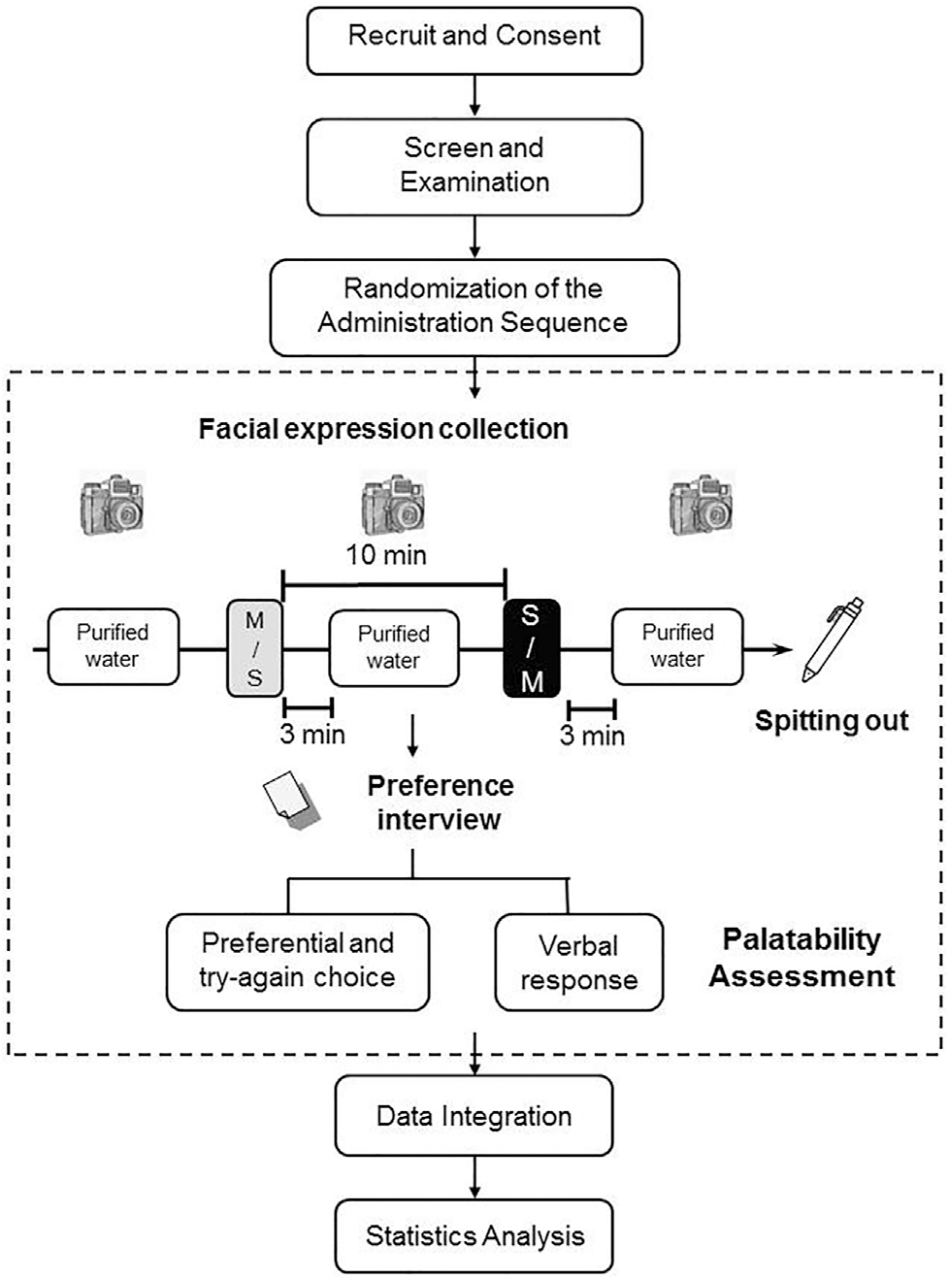
Flowchart of this study.

Forty-two child participants, 19 girls (mean age 7.6 ± 2.7 years) and 23 boys (mean age 6.3 ± 2.9 years), were enrolled in this study (the flowchart in Figure 2). Twenty children first tasted the carbocysteine oral solution with mint taste and then the preparation with strawberry taste (M-S sequence), while 22 children tasted the strawberry preparation first and then the mint one (S-M sequence).

The emotional valence of mint preparation (−0.9 in M-S sequence and −1.2 in S-M) was both relatively lower than that of strawberry’s (both −0.7 in M-S and S-M) in two sequence groups. Using the crossover analysis method, the carry-over effect and the treatment effect did not affect the difference between the emotional valences of mint and strawberry preparation (*p* > 0.05).

On the other hand, the preference rates of strawberry preparation were higher than those of mint’s (Mailand-Gart test, *p* < 0.05). Similarly, the occurrence rate of “spitting out” for mint preparation was higher than that for strawberry preparation (Mailand-Gart test, *p* < 0.05). Using the mixed effect model to consider the effects of administration sequence and time, we could also observe the preparation effect on the emotional valences. The univariate and multivariate analysis results are listed in Table 1.

**TABLE 1:** The comparisons of emotional valences between two administration sequences of carbocysteine oral solution for mint taste (M) and strawberry taste (S).

	M-S	S-M	*p*-value
Emotional valence			
The 1st preparation	−0.9 (−1.2, −0.4)	−0.7 (−1.4, −0.3)	NA
The 2nd preparation	−0.7 (−1.0, −0.3)	−1.2 (v1.5, v0.7)	NA
Sum of EV	−1.5 (−1.9, v0.6)	−2.2 (−2.9, −0.5)	0.296
Difference of EV	0.2 (−0.1, 0.5)	0.3 (−0.3, 0.7)	0.641

Children randomized in the M-S sequence first tasted the carbocysteine oral solution mint taste and then the strawberry preparation, and children in the S-M sequence first tasted the strawberry preparation and then that for mint taste. NA indicated not applicable compared directly using statistic test. The sum of EV represented the carry-over effect, and the difference of EV (S minus M) indicated the treatment effect. The interaction analysis for the crossover design showed that the administration sequence and administration times were not statistically significant (*F* = 0.105 and 0.130), whereas only preparation was significant with *F* = 7.210 (*p* = 0.011) using the mixed effect model.

**FIGURE 3:**
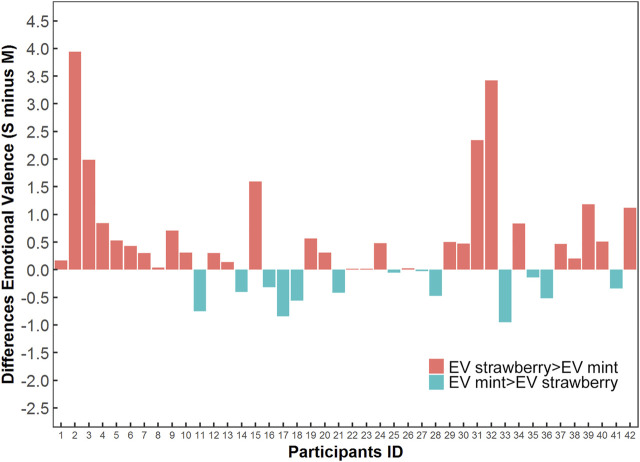
The distributions and differences of the emotional valence for child participants taking carbocysteine preparations.

The emotional valences for mint preparation were higher than those of strawberry taste in 13 participants (31%); 69.0% (29/42) of participants’ emotional valences for strawberry preparation were higher (Figure 3). The difference in emotional valences between the two preparations was significant by Wilcoxon signed rank test (Supplementary Figure S1). Among 27 participants aged ≥5 years, the taste preference rate was 88.5% (23/26) for the children’s preparation (one missing value for the taste preference), and 77.8% of participants (21/27) chose the children’s preparation if they had to take the medicine one more time. The taste and try-again preference among the children are shown in Table 2.

**TABLE 2:** The comparisons of spit-out and taste interviews between two administration sequences of carbocysteine oral solution for mint taste (M) and strawberry taste (S).

	M-S	S-M	*p*-value
Spitting out			
The 1st preparation	7 (77.8)	0	0.007
The 2nd preparation	2 (22.2)	6 (100)	
Taste preference			
The 1st preparation	2 (14.3)	11 (91.7)	<0.001
The 2nd preparation	12 (85.7)	1 (8.3)	
Try-again preference			
The 1st preparation	3 (21.4)	10 (76.9)	0.007
The 2nd preparation	11 (78.6)	3 (23.1)	

Children randomized in the M-S sequence first tasted the carbocysteine oral solution mint taste and then the strawberry preparation, and children in the S-M sequence first tasted the strawberry preparation and then that for mint taste.

No adverse event was observed in this study.

## Discussion

Carbocysteine is a mucolytic drug that acts by disrupting disulfide bridges between macromolecules, leading to reduced mucus viscosity in the respiratory tract. This drug is widely used to treat pediatric acute coughs, bronchitis, and asthma in many countries (Zeng et al., 2017; Skiendziel et al., 2019). The taste and palatability of medications play a pivotal role in ease of administration and effective therapeutic outcomes for pediatric patients. According to a survey, pediatric medications accounted for less than 10% of the total number of medications, and more than 90% of medications used in children were not initially developed for the pediatric population (Li et al., 2015). Before the carbocysteine oral solution strawberry taste (100 ml: 2 g) was marketed in China, the clinical effects of carbocysteine, which was only developed for adult usage with mint taste, were limited due to the taste sensitivity and unacceptability of children. This is an even more critical issue in the development of pediatric pharmaceutical products. Effective drugs that do not taste bitter will greatly improve medication compliance in children. How to effectively evaluate the palatability of pediatric pharmaceutical products is therefore important to better the taste of medications. In this study, a facial action coding system, expression recognition, and analysis technology were expanded and applied in the palatability assessment of the two carbocysteine preparations. Results confirmed that flavor masking technique could improve the palatability of pediatric pharmaceutical products. Moreover, this study showed that it is crucial to have scientific and feasible procedures of palatability assessment for pediatric pharmaceutical products.

Palatability assessment for two carbocysteine preparations was conducted through a comprehensive approach, including facial action coding system, spitting out, taste and try-again preferential method, and verbal response by interview. The results of this study indicated that carbocysteine oral solution strawberry taste was superior to mint preparation in taste and try-again preference, spitting out, and emotional valence. For carbocysteine medication, previous studies found that carbocysteine cough syrups were well tolerated in children over 2 years of age (Cohen et al., 2017) and combining carbocysteine powder formulation with yogurt could improve the palatability of carbocysteine for children over 2 years old (Tanaka et al., 2020b). Few comparative studies illustrated the palatability of carbocysteine oral solution with flavor masked technique. From the methodological aspect, most studies (Anand et al., 2008; Salman et al., 2018b) adopt the scaling methods, and some researchers have developed a novel hedonic taste scale for pediatric use (Wagner et al., 2020). These are mainly based on the Likert scale with some cartoons in order to facilitate children’s understanding. Other recommended methods include ranking order or preferential, facial expressions, and verbal feedback (Anand et al., 2008; Ameen et al., 2016).

At present, most studies on the evaluation of children’s drug taste or palatability mostly adopt the scale method, and some researchers have developed corresponding evaluation scales for young children, mainly drawing cartoons of Likert scale to facilitate understanding for children. Other recommended methods include ranking or bias evaluation, facial action coding system, and verbal response. Among these, the advantage of facial action coding system is suitable for non-verbal children, e.g., infants (Mennella et al., 2013). More importantly, the algorithm can be used for quantitative analysis of the emotional valence of children’s facial expressions when taking the drug, but this depends on the algorithm’s accuracy. The limitations of this study relate to the establishment of basic data and parameters of children’s facial expressions. Assessing facial expressions without shielding, especially for young children, is also crucial.

Furthermore, from the study design of this palatability assessment, the crossover design could save the sample size and overcome the bias caused by individual variations in taste sensitivity. It is necessary to analyze and interpret the results through the crossover design statistical analysis method. In this study, regardless of the results of univariate and multivariate analysis, it was suggested that different sequences and times of administration (M-S vs. S-M) had no effect on the primary outcome of palatability assessment, the emotional valence, as well in other indicators such as spitting rate, taste preference, and try-again preference. The differences of the above outcomes only reflected the differences between preparations. This provided a typical example of design and statistical analysis for pediatric pharmaceutical products of palatability assessment.

It should be emphasized that better palatability is only one aspect of improving medication compliance for pediatric pharmaceutical usage. For palatability assessment, *in vitro* research methods, such as the electronic tongue method, have strong applicability in early evaluation. Thus, to solve the problem of medication compliance for pediatric pharmaceutical usage, it is necessary to comprehensively consider several factors involved in pediatric pharmaceutical research and development. Often, even though a preparation revealed good palatability in a clinical trial, this finding might not be reproduced when children consume the product in everyday environments such as home or school. Ultimately, this complicates the validity of palatability assessment in a clinical trial scenario.

This study provided a promising idea for the methodology and a specific practical case for pediatric pharmaceutical products of palatability assessment. Nevertheless, there were still some limitations that needed to be considered. Firstly, due to the era of the coronavirus disease 2019 (COVID-19) pandemic, and considering the prevention and control requirements of COVID-19, this single-center trial involved healthy children volunteers. Although in the design phase, we planned to recruit both healthy volunteers and hospitalized patients who were indicated to be prescribed carbocysteine; hospitalized patients were not included. Potential differences in taste perception between healthy children and patients may influence palatability assessment results, which needs to be further explored in future studies to clarify consistency in patient taste evaluation. Secondly, FaceReader™, the software used to collect and analyze children’s facial expressions when taking medications, was developed and applied for adults in non-medical scenarios. It still lacked the basic data and parameters of children facial expressions, although facial expressions from five healthy children at different ages were collected to establish facial parameters of children with small samples in the pilot stage. Facial sampling in children in the pilot stage involved recording 3–5 min of natural facial expression and was calibrated to the child’s neutral face, prior to administering each tastant, to minimize bias towards a specific set of facial characteristics. The palatability assessment was comprehensively evaluated by series tools, including facial action coding system, preferential and try-again method, and verbal response by interview. The measurement results of those tools can be used as a reference for cross-validation to some extent. It suggested that a facial action coding system could be used for the palatability evaluation in children. In further research and development, children’s facial parameters and fundamental data should be established through large sample collection to facilitate the refinement and accuracy of facial expression analysis.

## Conclusion

The carbocysteine oral solution strawberry taste is an appealing preparation for clinical use since it was better received by children. This formulation may facilitate ease of administration and compliance in young pediatric patients over 2 years of age. The facial action coding system and professional analysis software could be a potential alternative for palatability assessment for pediatric pharmaceutical products.

## Data Availability

The raw data supporting the conclusion of this article will be made available by the authors, without undue reservation.

## Supplementary Material

Supplementary Figure S1 The distributions and paired test for emotional valences of two preparations
