# Supplementary figures and images for: Palatability Assessment of Carbocysteine Oral Solution Strawberry Taste Versus Carbocysteine Oral Solution Mint Taste: A Blinded Randomized Study
Source: Front Pharmacol. 2022 Feb 28;13:822086. doi: 10.3389/fphar.2022.822086 (PMC8919395; doi:10.3389/fphar.2022.822086)

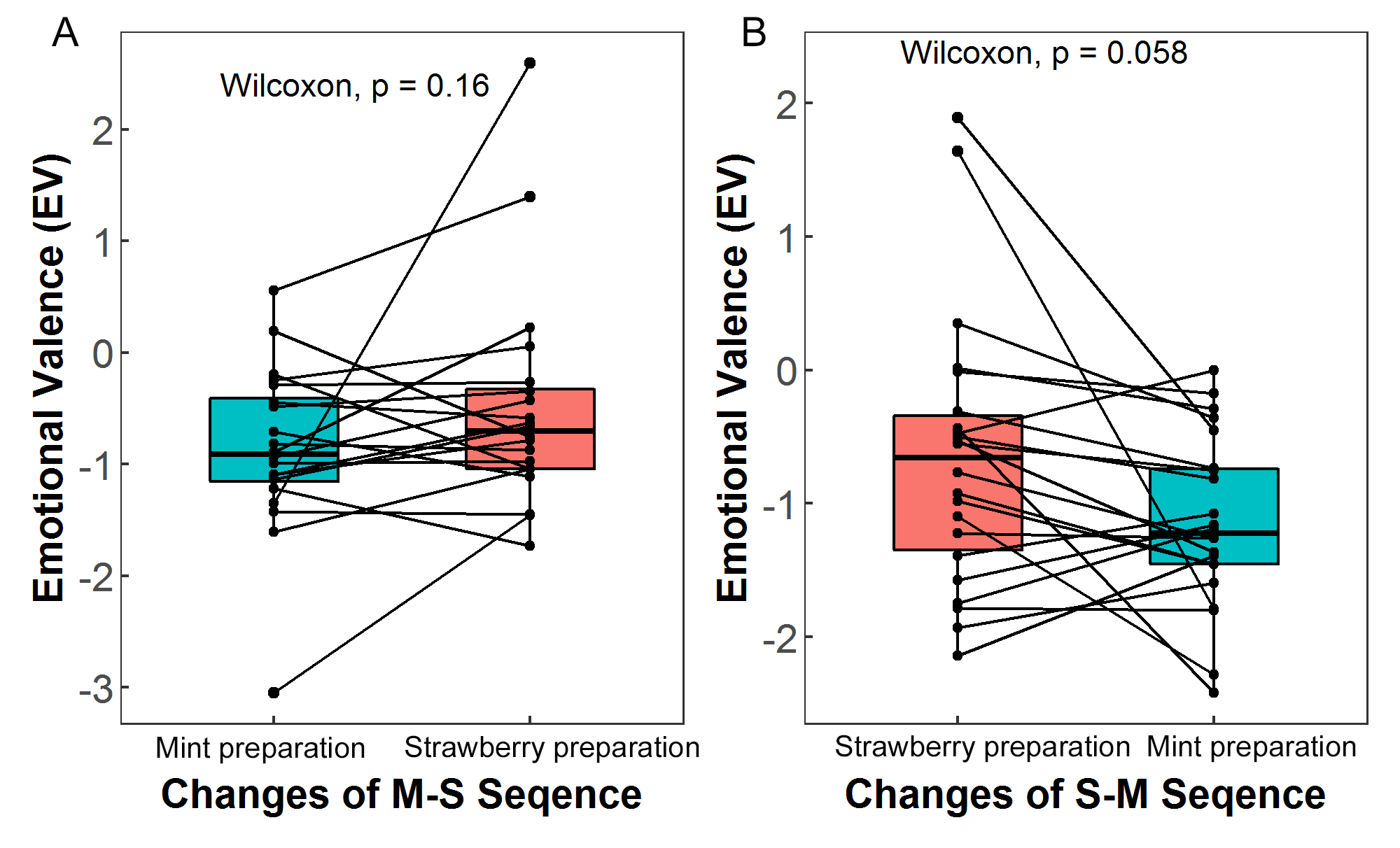

Supplement: Supplementary file 1 [file Image1.TIFF]
